# Metformin alleviates osteoarthritis in mice by inhibiting chondrocyte ferroptosis and improving subchondral osteosclerosis and angiogenesis

**DOI:** 10.1186/s13018-022-03225-y

**Published:** 2022-06-28

**Authors:** Jiangbo Yan, Gangning Feng, Long Ma, Zhirong Chen, Qunhua Jin

**Affiliations:** 1 Ningxia Medical University, The General Hospital of Ningxia Medical University, Yinchuan, Ningxia China; 2 Orthopedics Ward 3, The General Hospital of Ningxia Medical University, Yinchuan, Ningxia China

**Keywords:** Osteoarthritis, Chondrocytes, Metformin, Ferroptosis, Subchondral osteosclerosis, Angiogenesis

## Abstract

**Background:**

Osteoarthritis (OA) is the most common musculoskeletal disease, and it has a complex pathology and unknown pathogenesis. Chondrocyte ferroptosis is closely associated with the development of OA. As a common drug administered for the treatment of type 2 diabetes, metformin (Met) is known to inhibit the development of ferroptosis. However, its therapeutic effect in OA remains unknown. The present study aimed to explore the effects of Met on cartilage and subchondral bone in a mouse OA model and to explore the potential underlying mechanisms.

**Methods:**

A mouse OA model was induced using destabilization of the medial meniscus (DMM) surgery, chondrocyte ferroptosis was induced using an intra-articular injection of Erastin, and Met (200 mg/kg/day) was intragastrically administered for 8 weeks after surgery. H&E and Safranin O‑fast green staining were used to evaluate cartilage degeneration, and μ‑computed tomography was used to evaluate changes in subchondral bone microarchitecture. Moreover, immunohistochemical staining was performed to detect mechanistic metalloproteinases 13, type II collagen, glutathione peroxidase 4, acyl-CoA synthetase long-chain family member 4, solute carrier family 7 member 11 and p53. Runt-associated transcription factor 2 and CD31 were detected using immunofluorescent staining.

**Results:**

Met protected articular cartilage and reversed the abnormal expression of ferroptosis-related proteins in the chondrocytes of DMM mice. Moreover, intra-articular injection of Erastin induced ferroptosis in mouse chondrocytes, and Met eliminated the ferroptosis effects induced by Erastin and protected articular cartilage. In addition, the results of the present study demonstrated that Met alleviated the microstructural changes of subchondral osteosclerosis and reduced heterotypic angiogenesis in DMM mice.

**Conclusion:**

Met alleviates the pathological changes of OA by inhibiting ferroptosis in OA chondrocytes, alleviating subchondral sclerosis and reducing abnormal angiogenesis in subchondral bone in advanced OA.

## Background

Osteoarthritis (OA) is the most common musculoskeletal disease affecting middle-aged and elderly patients worldwide [1] and is mainly characterized by degeneration, destruction and hyperosteogeny of the knee cartilage [2]. An increasingly aging population is causing an increasing economic burden to patients, families and society [3, 4]. The main pathological features of OA include degeneration of articular cartilage, synovitis and subchondral osteosclerosis, while pathological death of chondrocytes due to trauma and abnormal mechanical stress is a key cause of articular cartilage degeneration [5]. Therefore, alleviating articular cartilage degeneration by inhibiting chondrocyte death remains the focus of current research. However, current therapeutic measures targeting only articular cartilage degeneration are limited, thus limiting the current understanding of OA pathology [6]. Articular cartilage and subchondral bone complement each other to maintain the normal function of the knee joint [7]. Results of a previous study demonstrated that changes in subchondral bone microarchitecture may affect the integrity of overlying cartilage, causing pathological changes simultaneously, or prior to articular cartilage degeneration [8, 9]. This indicates that therapeutic measures for targeting the pathological changes of OA subchondral bone are of great importance.

Ferroptosis, as a mode of cell death, was initially proposed by Dixon et al. [10] in 2012. Erastin, as a specific activator of ferroptosis, inactivates glutathione (GSH) peroxidase 4 (GPX4) by inhibiting the import of cystine, causing intracellular lipid peroxidation and the accumulation of reactive oxygen species, ultimately leading to the occurrence of ferroptotic events [11, 12]. Numerous previous studies have demonstrated that GPX4 is a key regulator of ferroptosis [12–14]. Moreover, acyl-CoA synthase long-chain family member 4 (ACSL4) is not only a sensitive monitor of ferroptosis, but also an important contributor to ferroptosis [15]. System Xc- is a cysteine-glutamate reverse transporter that plays an important regulatory role in the GPX4 pathway in ferroptosis. As a specific subunit of system Xc-, solute carrier family 7 member 11 (SLC7A11) inhibits the occurrence of ferroptosis by inhibiting intracellular GSH accumulation and lipid peroxidation [16]. The results of previous studies have demonstrated that the tumor suppressor p53 is also a key modulator of ferroptosis, which targets and inhibits SLC7A11 expression, and sensitizes cells to ferroptosis [17]. Ferroptosis plays a major role in numerous chronic degenerative diseases, including ischemia–reperfusion injury, cerebral ischemia, stroke and cancer [11]. The results of a further previous study demonstrated that ferroptosis in chondrocytes increased the destruction of articular cartilage and accelerated the progression of OA [18].

During the development of OA, the subchondral bone adapts to abnormal mechanical stress, mainly through bone remodeling [19]. Bone remodeling in the early stage is characterized by increased bone loss and decreased bone mineral density, and in the late stage, it is often characterized by increased bone mineral density, subchondral bone plate, bone thickness and bone formation [20, 21]. Subchondral osteosclerosis is an important pathological marker of OA [22]. The results of our previous study demonstrated that a mouse OA model induced by surgery developed late pathological changes with subchondral osteosclerosis 8 weeks after surgery [23, 24]. The specific mechanisms underlying subchondral osteosclerosis in advanced stages of OA remain to be fully elucidated. Both abnormal bone remodeling and neovascularization are observed in OA subchondral bone; notably, neovascularization invades the upper cartilage layer and accelerates cartilage destruction [25, 26], and it also stimulates the production of inflammatory mediators in the articular cartilage, stimulates articular cartilage innervation and causes joint pain [27]. However, the discovery of novel drugs for the abnormal neovascularization of the subchondral bone is required.

Metformin (Met) is a commonly used drug administered for the treatment of type 2 diabetes, and it exerts numerous positive effects on a variety of degenerative diseases, including cardiovascular and cerebrovascular diseases, chronic kidney disease and aging [28]. Moreover, it may also exert anti-angiogenic effects in a variety of tissues, including granulation tissue, retinal vascular endothelium and tumor cells [29–31]. The results of a prospective study demonstrated a significantly reduced rate of medial compartment lesion of knee OA within 4 years, and a significantly reduced risk of total knee arthroplasty within 6 years following treatment with Met in obese patients, compared with patients who were not administered Met [32]. These results suggested that Met may delay disease development in OA. The results of our previous study demonstrated that Met exerted chondroprotective effects by inhibiting chondrocyte pyroptosis in a mouse OA model [23]. However, the effects of Met on ferroptosis in different tissue cells remain inconclusive, and the results of a previous study indicated that Met may affect the pathological progression of hyperlipidemia-related vascular calcification through anti-ferroptosis in vascular smooth muscle cells [33]. Moreover, the results of a different study demonstrated that Met may inhibit the growth of tumor cells by inducing the occurrence of ferroptosis in tumor tissues [34]. To date, the specific role of Met in ferroptosis in OA chondrocytes has not been reported, and whether Met exerts anti-angiogenic effects in OA subchondral bone remains to be fully elucidated. Thus, a destabilization of the medial meniscus (DMM)-induced OA mouse model was established in the present study, and the mechanisms by which Met affected OA progression were explored in vivo.

## Materials and methods

### Animals

**Fig. 1:**
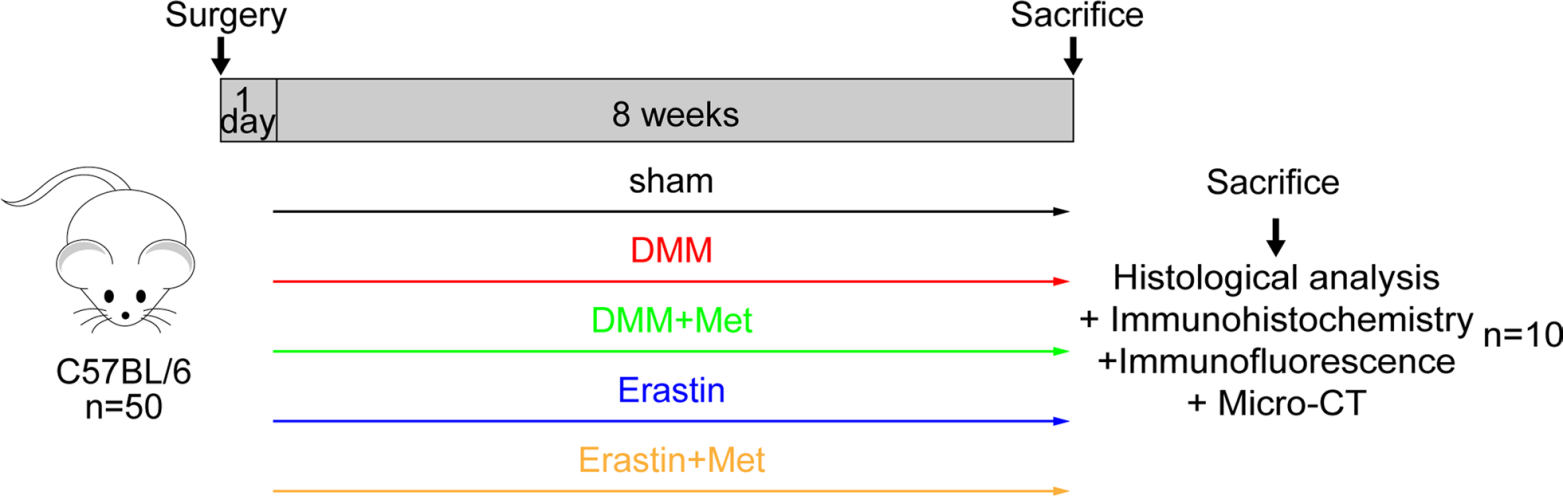
Schematic diagram of experimental design

A total of 50 healthy, wild-type adult male C57BL/6 mice (age, 8 weeks; weight 20–25 g) were purchased from the Laboratory Animal Center of Ningxia Medical University. Mice had free access to food and water and the experimental environment was SPF grade (temperature, 22 ± 1 °C; humidity, 55%; light/dark cycle, 12/12 h). The experiment was approved by the Animal Ethics Committee of Ningxia Medical University (Yinchuan, China; protocol no. 2020–115). Animal experiments were carried out in accordance with the Arrive guidelines [35]. A schematic demonstrating the experimental protocol is displayed in Fig. 1.

A total of 50 mice were randomly divided into five groups (*n* = 10 in each group): Sham, DMM, DMM + Met, Erastin, and Erastin + Met groups. In order to establish a mouse OA model, the right knee of mice was selected for DMM surgery [36]. Briefly, after mice were anesthetized with an intraperitoneal injection of 1% sodium pentobarbital (60 mg/kg) [37], the meniscotibial ligament of the right knee was transected to free the anterior crus of the meniscus, and sham surgery was performed without transecting the meniscotibial ligament. Mice were injected intra-articularly with Erastin (15 mg/kg) [38], and the sham and DMM groups were injected with an equal volume of saline twice a week. Moreover, Met was administered by gavage (200 mg/kg/day) [23]. Mice were killed by overdose anesthesia 8 weeks after surgery, and the right knee joint was collected for subsequent experiments.

### μ‑computed tomography (CT)

The right knee joints of mice were fixed with 4% paraformaldehyde at room temperature for 48 h after removal of the surrounding skin and muscle and scanned with a μ-CT scanner (SkyScan 1176; Kontich Belgium) at a resolution of 9 μm/pixel, an exposure time of 900 ms, a voltage of 50 kV and a current of 500 µA. Data analysis was performed using CTAn (Bruker MicroCT, Kontich, Belgium) and DataViewer (Bruker MicroCT, Kontich, Belgium). Images were reconstructed using NRecon (Bruker MicroCT, Kontich, Belgium). The region of interest was between the tibial subchondral plate and the tibial articular cartilage surface, and the indicators included: bone volume fraction (BV/TV, %), trabecular separation (Tb. Sp, mm) and bone mineral density (BMD, g/cm^3^).

### Histological assessment

Following μ-CT analysis, knee tissues were rinsed in PBS at room temperature for 3 h. (Rinses were changed every 30 min.) 10% EDTA was used for decalcification at room temperature for 2 weeks. Tissues were embedded in paraffin following graded ethanol dehydration processing. The thickness of the coronal section of the knee was 4 μm, and this was stained using H&E (Cat. no. G1005; Wuhan Servicebio Technology Co., Ltd.), according to the manufacturer’s protocol. Briefly, deparaffinization was performed twice for 20 min using 100% xylene and twice for 10 min using 100% ethanol at room temperature. Immersion was carried out using 75% ethanol for 5 min, followed by staining with hematoxylin for 5 min at room temperature. Subsequently, samples were rinsed in tap water for 15 min, 85 and 95% ethanol once for a total of 10 min, and stained with eosin for 5 min at room temperature. For Safranin O-fast green staining (Cat. no. G1053; Wuhan Servicebio Technology Co., Ltd.), the section deparaffinization process was as previously described. According to the manufacturer’s protocol, sections were stained using fast green for 6 min at room temperature, and following dehydration, samples were stained in safranin for 3 min at room temperature. Degeneration of cartilage was assessed using a blinded method and scored using the Osteoarthritis Research Society International (OARSI) score [39]. Measures included: thickness of hyaline cartilage (HC; distance from tidal line to articular cartilage surface) and thickness of calcified cartilage (CC; distance from tidal line to subchondral bone plate). A total of five random fields in three random sections per mouse were observed using an Olympus DP71 light microscope (Olympus Corporation). Image-Pro Plus version 6.0 software (Media Cybernetics, Inc.) was used to perform the statistical analysis.

### Immunohistochemistry and Immunofluorescence

The thickness of the sagittal serial sections of the paraffin-embedded mouse knee joints was 4 μm. Sections were deparaffinized and hydrated as previously described, and antigen retrieval was performed using 0.1% trypsin for 30 min at 37 °C, followed by blocking of endogenous peroxidase with 3% hydrogen peroxide for 10 min. After blocking for 30 min at 37 °C with 5% normal goat serum (OriGene Technologies, Inc.), samples were incubated with the following primary antibodies overnight at 4 °C: Anti-MMP‑13 (cat. no. ab39012; 1:300; Abcam), anti‑Col II (cat. no. ab34712; 1:300; Abcam), anti‑GPX4 (cat. no. 67763-1-Ig; 1:200; ProteinTech Group, Inc.), anti‑ACSL4 (cat. no. ab155282; 1:300; Abcam), anti‑SLC7A11 (cat. no. 26864–1-AP; 1:200; ProteinTech Group, Inc.), anti‑p53 (cat. no. 60283-2-Ig; 1:200; ProteinTech Group, Inc.), anti‑Runx2 (cat. no. ab192256; 1:300; Abcam), anti‑CD31 (cat. no. ab9498; 1:300; Abcam). For immunohistochemical staining, a ZSGB-Bio kit (OriGene Technologies, Inc.) was used according to the manufacturer’s instructions. Briefly, samples were incubated in reaction enhancement solution (reagent 1) for 30 min at 37 °C and enhancement enzyme-labeled goat anti-rabbit IgG polymer (reagent 2) for 30 min at 37 °C. The color was developed using 3,3’-diaminobenzidine (DAB; ZSGB-Bio; OriGene Technologies, Inc.), and the nuclei were counterstained using hematoxylin(ZSGB-Bio; OriGene Technologies, Inc.). For immunofluorescent staining, sections were incubated with Alexa Fluor^®^ 488 goat anti-rabbit secondary antibody (cat. no. ab150077; 1:500; Abcam) for 1 h at 37 °C in a dark room. A total of five random fields in three random sections per mouse were observed using an Olympus DP71 light microscope (Olympus Corporation). Image-Pro Plus was used to perform the statistical analysis.

### Statistical analysis

All data are presented as the mean ± standard deviation. GraphPad Prism version 8.0 (GraphPad Software, Inc.) was used for statistical analysis. After the data were tested for homogeneity of variance, one-way ANOVA and Tukey’s multiple comparison tests were used to compare the data. Nonparametric data (OARSI scores) were analyzed using the Kruskal–Wallis H test, followed by a Dunn’s test. *P* < 0.05 was considered to indicate a statistically significant difference.

## Results

### Met attenuates cartilage degeneration in DMM mice

**Fig. 2:**
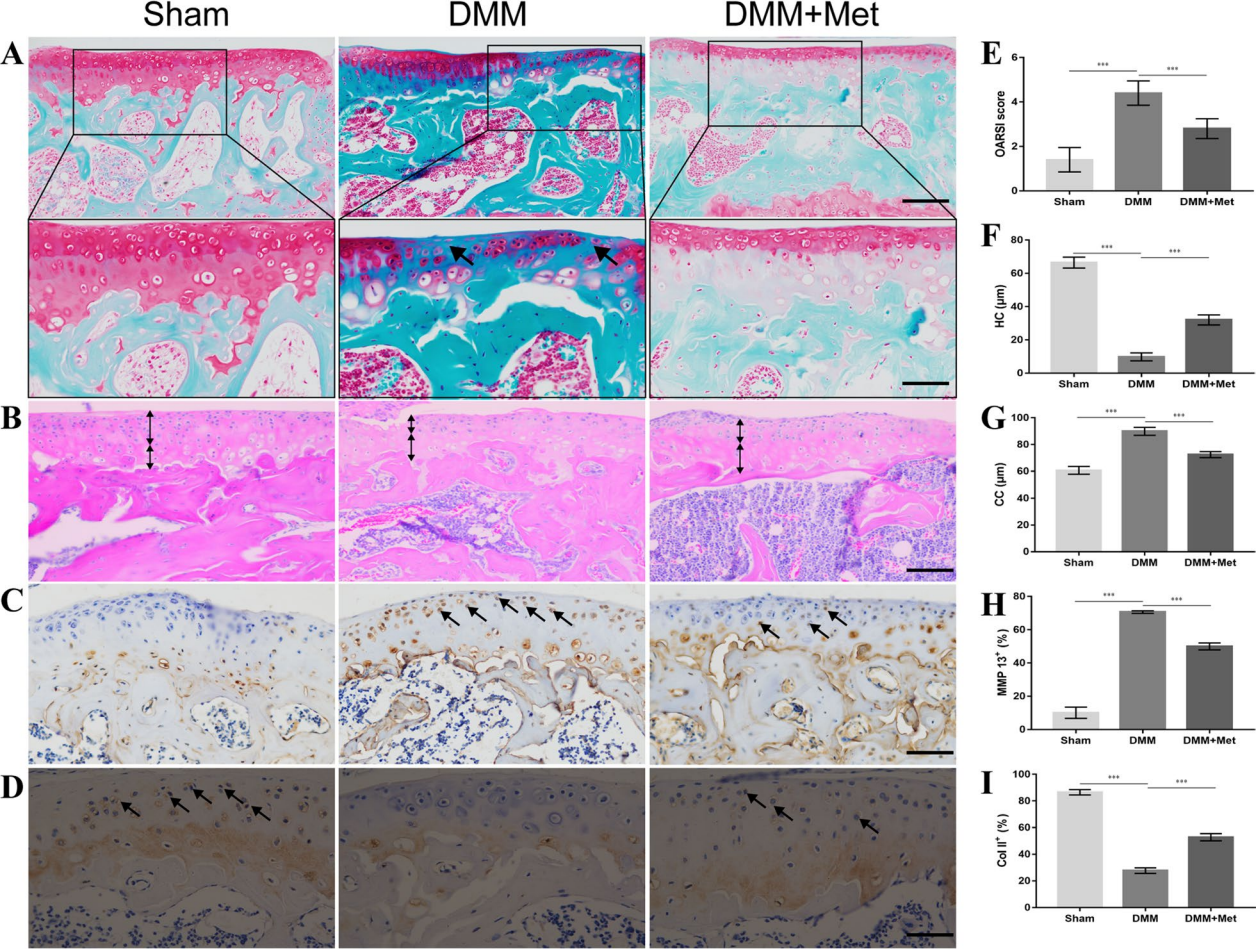
Metformin attenuates cartilage degeneration in the destabilization of the medial meniscus mice model. **A** Safranin O-fast green staining with arrows indicating cartilage proteoglycan loss (scale bar, 100 µm and 50 µm) and **E** OARSI score. **B** H&E staining (scale bar, 100 µm), black arrows indicate HC thickness and white arrows indicate CC thickness, and thickness of **F** HC and **G** CC. **C** MMP 13 and **D** Col II immunohistochemical staining (scale bar, 100 µm), arrows indicate positive cells, and **H** MMP13 and **I** Col II positive cell proportions. One-way ANOVA and Tukey’s multiple comparison tests were used to compare the data. Nonparametric data (OARSI scores) were analyzed using the Kruskal–Wallis H test followed by Dunn’s test. ***P < 0.001

To investigate the effects of Met on articular cartilage in DMM mice, the degree of cartilage degeneration was assessed by H&E, Safranin O-fast green and immunohistochemical staining. The results of the H&E staining demonstrated that mice in the DMM group exhibited significant cartilage degeneration 8 weeks after surgery, which primarily manifested as chondrocyte hypertrophy, discontinuous microcracks on the cartilage surface, thinning of the HC layer accompanied by thickening of the CC layer. These changes were partially recovered following treatment with Met (Fig. 2B, F and G). The results of the Safranin O-fast green staining demonstrated that the proteoglycans of articular cartilage were significantly decreased in mice in the DMM group at 8 weeks following surgery, and treatment with Met partially alleviated the loss of proteoglycans (Fig. 2A). These results were further verified using the OARSI score. The OARSI score was significantly higher in the DMM group compared with the sham group, and treatment with Met partially reduced the OARSI score (Fig. 2E). The results of the immunohistochemical analysis demonstrated increased expression of the catabolic marker MMP13 (Fig. 2C and H) and decreased expression of the anabolic marker Col II (Fig. 2D and I) in the DMM group compared with the sham group. The aforementioned changes returned to baseline levels following treatment with Met. These results indicated that Met may partially alleviate the degeneration of articular cartilage in DMM mice.

### Met attenuates cartilage degeneration induced by Erastin in mice

**Fig. 3:**
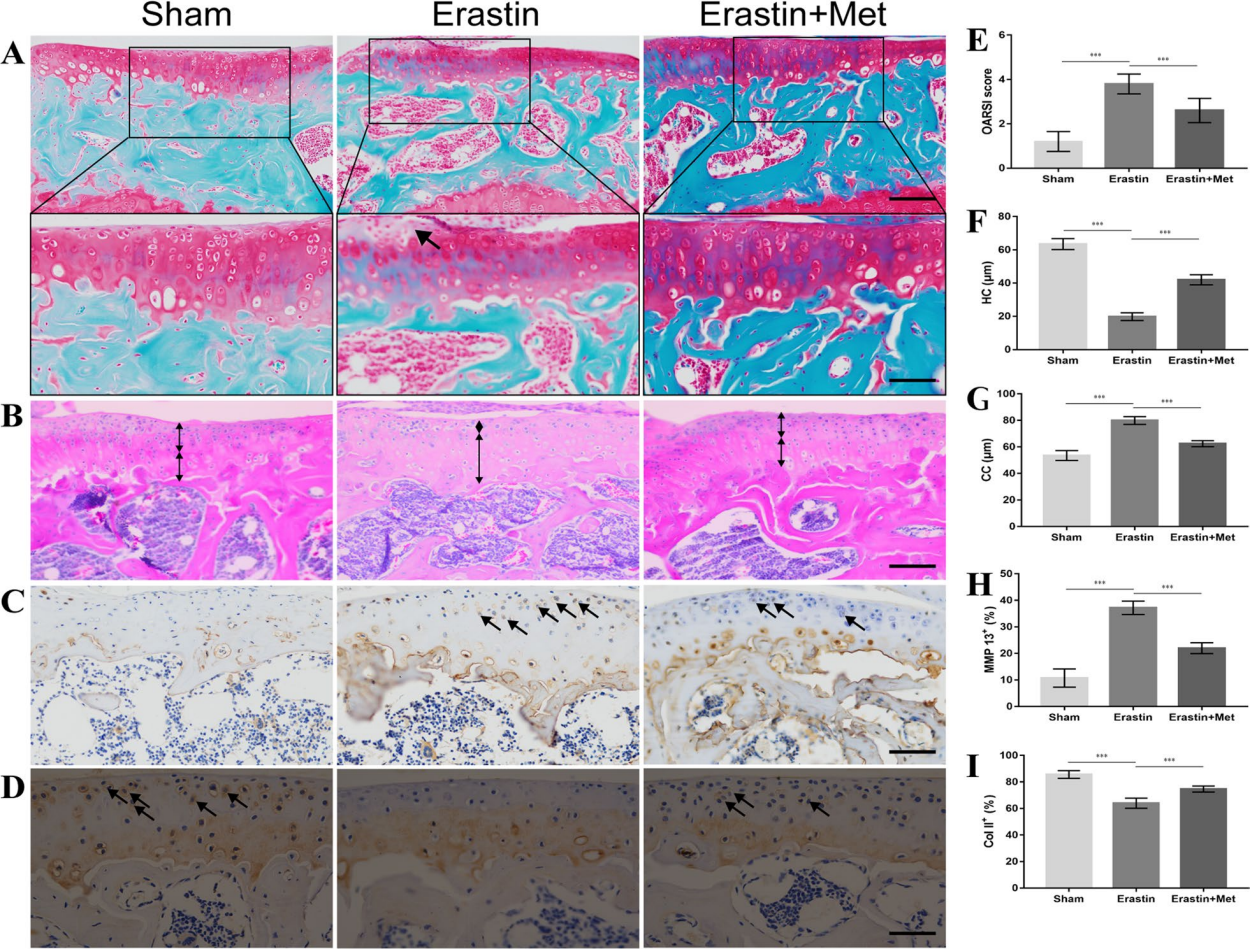
Metformin attenuates cartilage degeneration in Erastin-induced mice. **A** Safranin O-fast green staining with arrows indicating cartilage proteoglycan loss (scale bar, 100 µm and 50 µm) and **E** OARSI score. **B** H&E staining (scale bar, 100 µm), black arrows indicate HC thickness and white arrows indicate CC thickness, and thickness of **F** HC and **G** CC. **C** MMP 13 and **D** Col II immunohistochemical staining (scale bar, 100 µm), arrows indicate positive cells, and (H) MMP13 and **I** Col2 positive cell proportions. One-way ANOVA and Tukey’s multiple comparison tests were used to compare the data. Nonparametric data (OARSI scores) were analyzed using the Kruskal–Wallis H test followed by Dunn’s test. ***P < 0.001

To further investigate the effects of Met on articular cartilage following Erastin-activated ferroptosis, chondrocyte ferroptosis was activated using intra-articular injection of Erastin. The degree of cartilage degeneration was assessed using H&E, Safranin O-fast green and immunohistochemical staining. The results of the H&E staining demonstrated that significant cartilage degeneration and microcracks on the cartilage surface occurred 8 weeks after intra-articular injection of Erastin in mice, accompanied by a decrease in HC thickness and an increase in CC thickness. These changes were partially restored following treatment with Met (Fig. 3B, F and G). The results of the Safranin O-fast green staining results demonstrated that proteoglycans from articular cartilage were significantly lost 8 weeks after intra-articular injection of Erastin in mice, and treatment with Met partially alleviated proteoglycan loss (Fig. 3A). These results were also verified by the OARSI score. Notably, compared with the sham group, the OARSI score was significantly higher in the Erastin group, and treatment with Met partially reduced the OARSI score (Fig. 3E). Immunohistochemical analysis demonstrated that the expression levels of the catabolic marker MMP13 (Fig. 3C and H) and the anabolic marker Col II (Fig. 3D and I) were abnormal in the Erastin group compared with the sham group. Moreover, these changes returned to baseline following treatment with Met. These results indicated that Met partially alleviated the Erastin-induced degeneration of articular cartilage in mice.

### Met attenuates ferroptosis in the chondrocytes of DMM mice

**Fig. 4:**
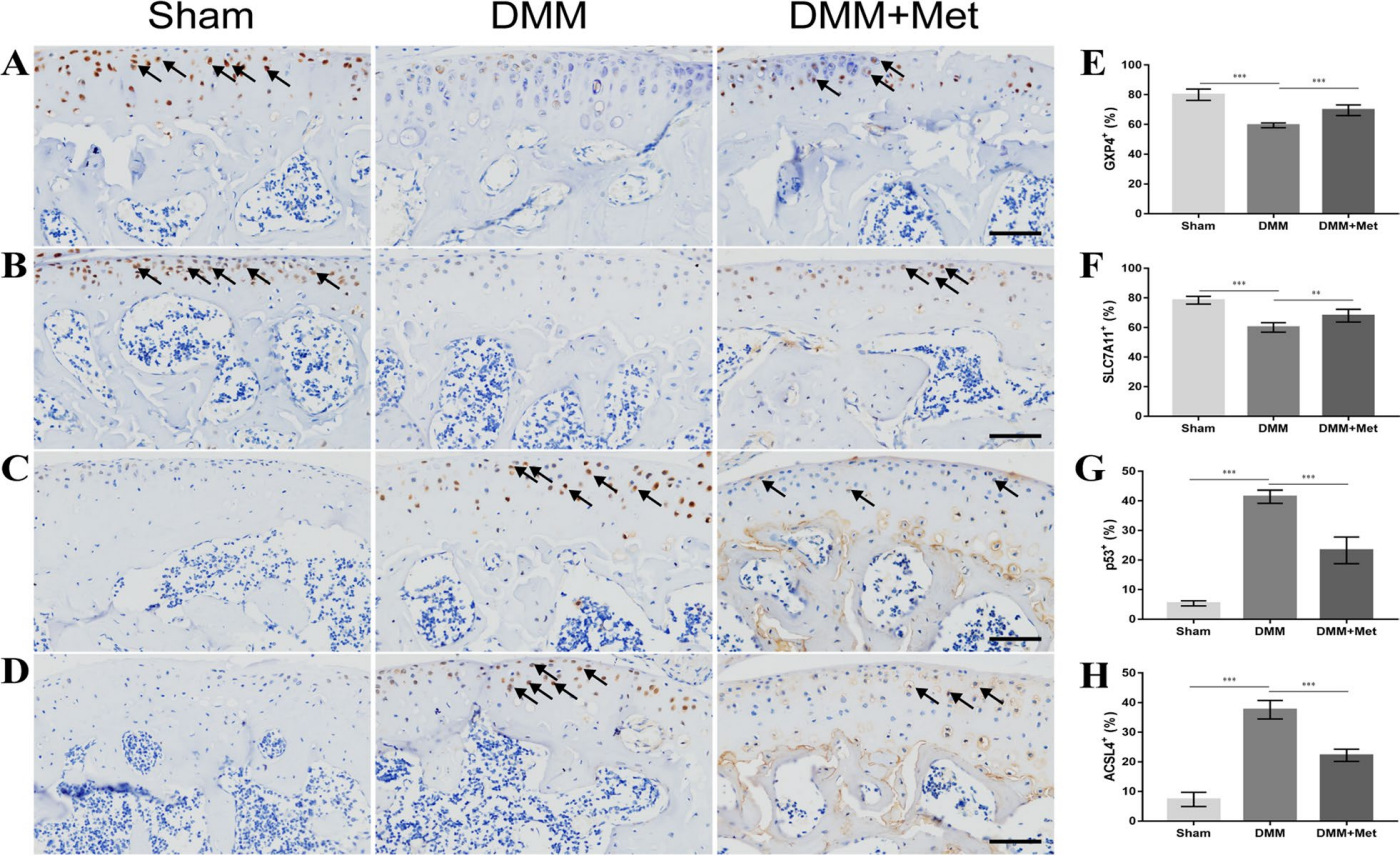
Metformin alleviated chondrocyte ferroptosis in the destabilization of the medial meniscus mice model. Immunohistochemical staining of **A** GPX4, **B** SLC7A11, **C** p53 and **D** ACSL4 (scale bar, 100 µm). Arrows indicate positive cells and **E** GPX4, **F** SLC7A11, **G** p53 and **H** ACSL4-positive cell proportions. One-way ANOVA and Tukey’s multiple comparison tests were used to compare the data. **P < 0.01; ***P < 0.001

To further verify the effects of Met on ferroptosis in the chondrocytes of DMM mice, immunohistochemical analysis was carried out. The results of the present study demonstrated the increased expression of ferroptosis-related parameters, including ACSL4 (Fig. 4D and H) and p53 (Fig. 4C and G), and the decreased expression of ferroptosis-related parameters, including GPX4 (Fig. 4A and E) and SLC7A11 (Fig. 4B and F) in the DMM group compared with the sham group. These changes were partially alleviated following treatment with Met. These results indicated that Met may alleviate ferroptosis in the chondrocytes of DMM mice.

### Met attenuates Erastin-induced ferroptosis in mouse chondrocytes

**Fig. 5:**
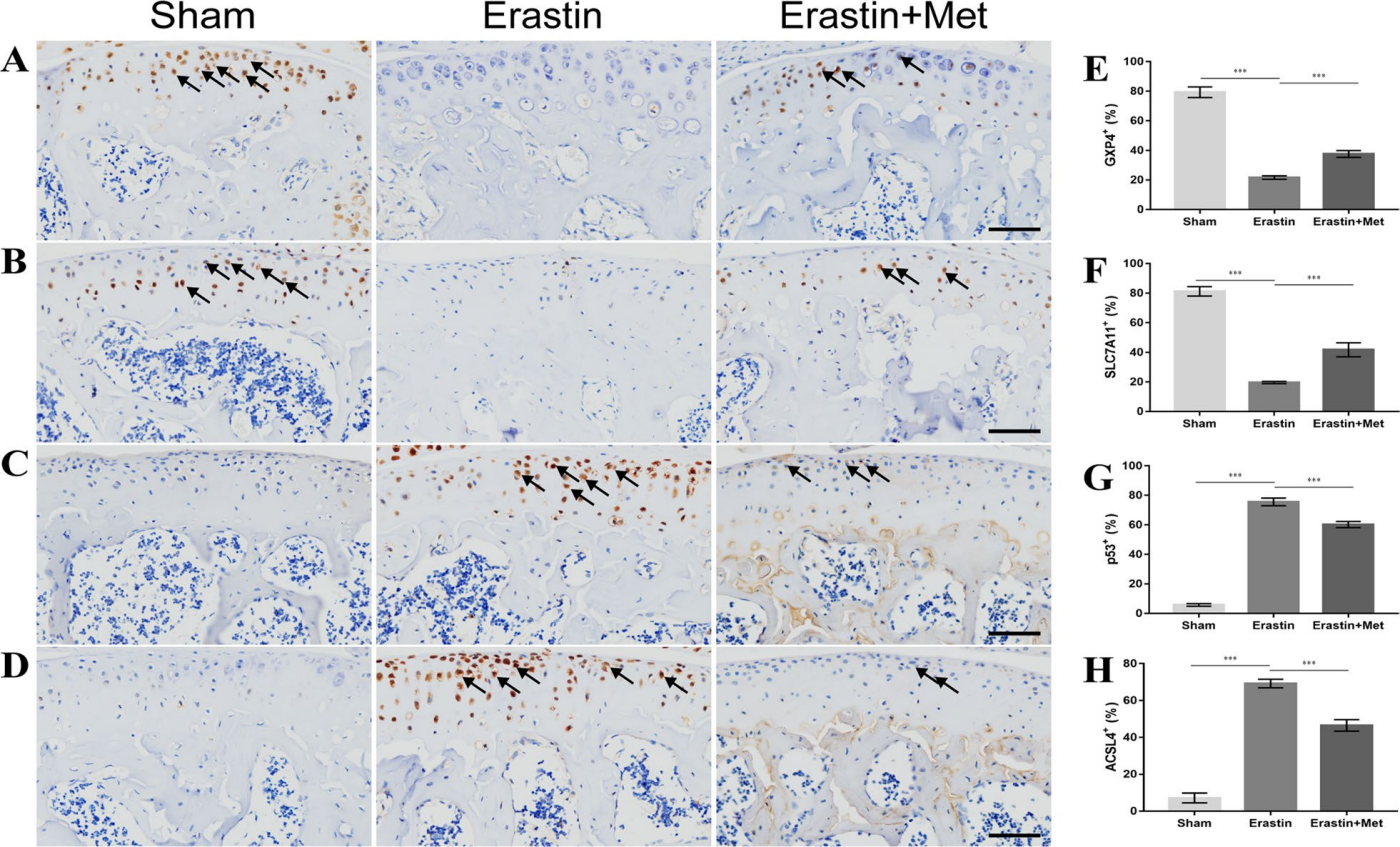
Metformin attenuates Erastin-induced ferroptosis in mouse chondrocytes. Immunohistochemical staining of **A** GPX4, **B** SLC7A11, **C** p53 and **D** ACSL4 (scale bar, 100 µm). Arrows indicate positive cells and **E** GPX4, **F** SLC7A11, **G** P53 and **H** ACSL4-positive cell proportions. One-way ANOVA and Tukey’s multiple comparison tests were used to compare the data. ***P < 0.001

Following the activation of chondrocyte ferroptosis using intra-articular injection of Erastin, the results of immunohistochemical analysis demonstrated that the changes in ferroptosis-related parameters, including the increased expression of ACSL4 (Fig. 5D and H) and p53 (Fig. 5C and G), and the decreased expression of GPX4 (Fig. 5A and E) and SLC7A11 (Fig. 5B and F) were more significant in the Erastin group compared with the sham group. Changes in ferroptosis-related parameters were significantly alleviated in the Met + Erastin group compared with the Erastin group. These results indicated that Met may attenuate Erastin-induced ferroptosis in chondrocytes.

### Met attenuates subchondral osteosclerosis in DMM mice

**Fig. 6:**
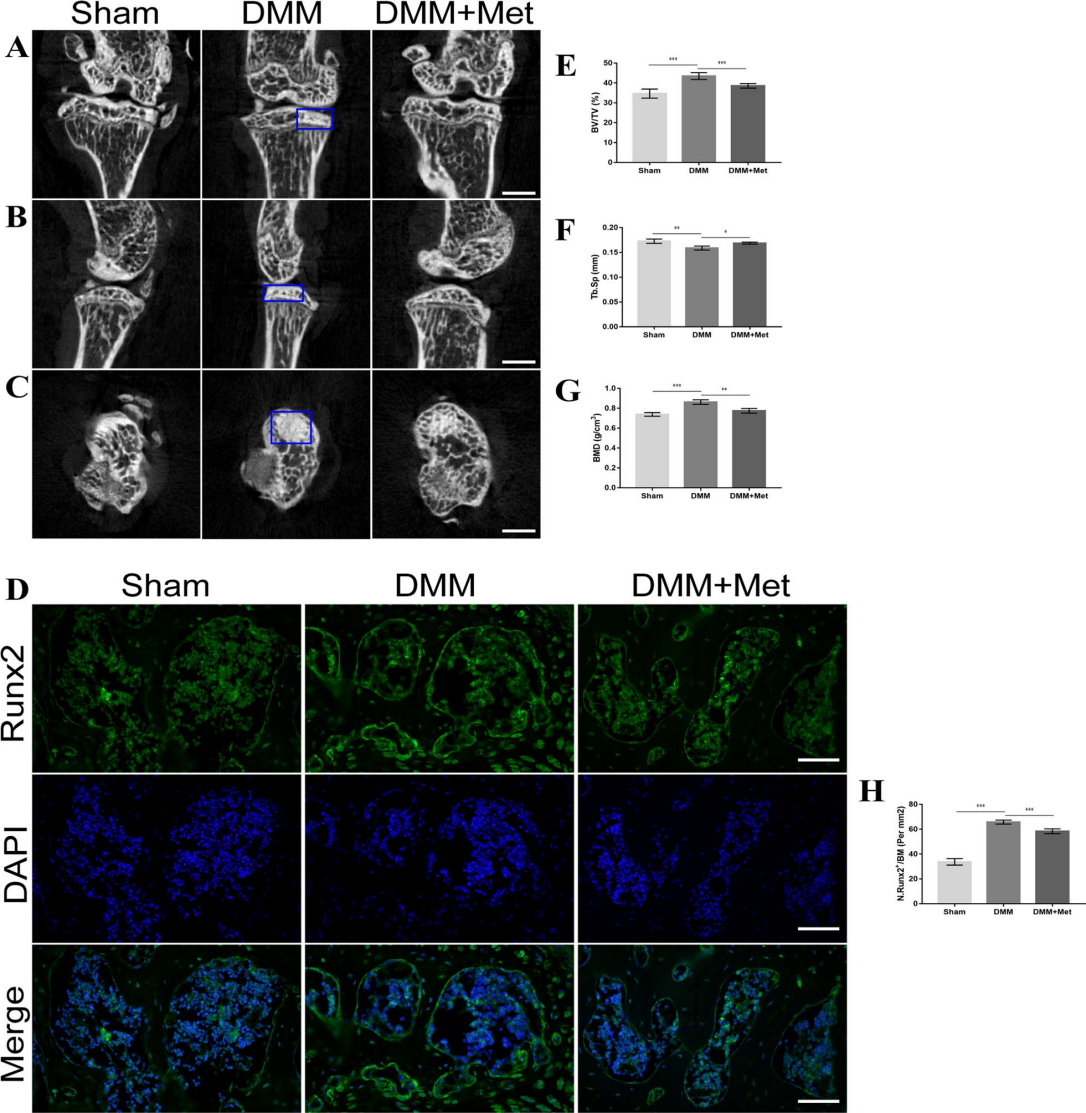
Metformin attenuates subchondral osteosclerosis in the destabilization of the medial meniscus mice model. **A** Sagittal plane, **B** coronal plane and **C** transaxial plane of μ-CT two-dimensional reconstruction, blue boxes indicating areas of osteosclerosis. **E** BV/TV, **F** Tb. Sp and **G** BMD. Immunofluorescence staining of **D** Runx2 and **H** the proportion of Runx2-positive cells (scale bar, 100 µm). One-way ANOVA and Tukey’s multiple comparison tests were used to compare the data. *P < 0.05; **P < 0.01; ***P < 0.001

To investigate the effects of Met on subchondral osteosclerosis in DMM mice, changes in subchondral bone-related parameters were assessed using μ-CT and immunofluorescence. Results of the μ-CT analysis demonstrated significant subchondral osteosclerosis 8 weeks after DMM surgery (Fig. 6A, B and C), highlighted by an increase in BV/TV (Fig. 6E) and BMD (Fig. 6G), and a decrease in Tb. Sp (Fig. 6F). Notably, treatment with Met alleviated sclerosis of the subchondral bone. Moreover, the results of the immunofluorescent staining demonstrated that the number of Runx2-positive cells in the subchondral bone of mice in the DMM group was significantly increased, compared with the control group. Compared with the DMM group, the number of Runx2-positive cells in the subchondral bone of mice in the DMM + Met group was significantly decreased (Fig. 6D and H). These results indicated that Met may alleviate subchondral osteosclerosis in DMM mice.

### Met attenuates subchondral bone angiogenesis in DMM mice

**Fig. 7:**
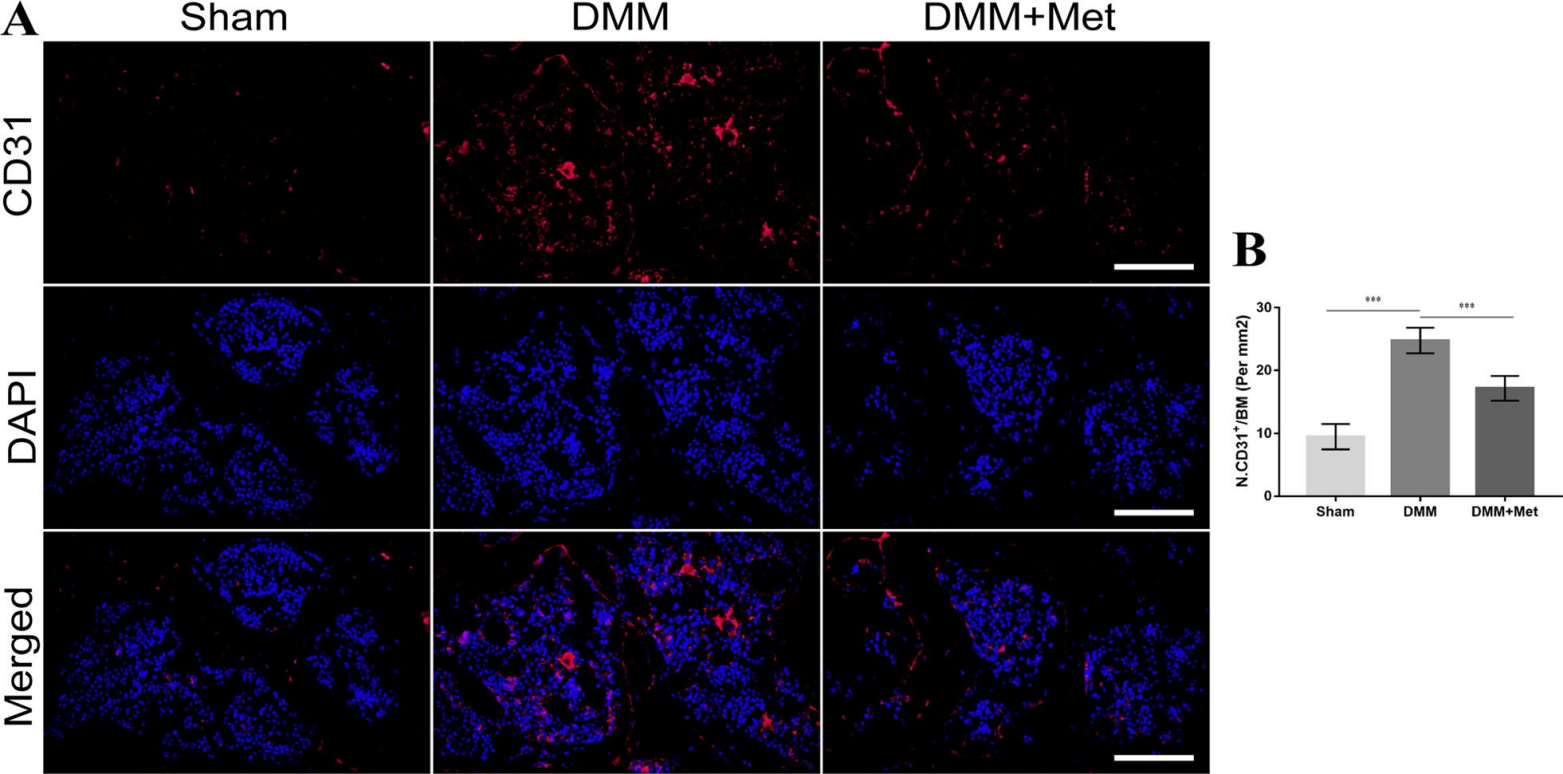
Metformin attenuates subchondral bone angiogenesis in the destabilization of the medial meniscus mice model. Immunofluorescence staining of **A** CD31 and **B** the proportion of CD31-positive cells (scale bar, 100 µm). One-way ANOVA and Tukey’s multiple comparison tests were used to compare the data. ***P < 0.001

To investigate the effects of Met on angiogenesis in the subchondral bone of DMM mice, the expression of the angiogenic marker CD31 was assessed using immunofluorescence. Compared with the control group, the expression of CD31 in the subchondral bone of mice in the DMM group was significantly increased. Compared with the DMM group, the expression of CD31 in the subchondral bone of mice in the DMM + Met group was significantly decreased (Fig. 7A and B). These results indicated that Met may alleviate angiogenesis in DMM mice.

## Discussion

OA, as the most prevalent joint disease, has been increasing year by year in recent years [40]. The results of previous studies have demonstrated that the death or survival of chondrocytes plays a key role in OA pathogenesis [41]. As a recently discovered mode of cell death, ferroptosis is associated with the pathological progression of numerous chronic degenerative diseases, such as ischemia–reperfusion injury, cerebral ischemia, stroke and cancer [11]. The results of previous studies confirm a specific association between ferroptosis and OA [42]. Thus, further investigations into the role of ferroptosis in the pathological progression of OA are required. As a common drug administered for the treatment of type 2 diabetes, Met has been demonstrated to target and inhibit the development of ferroptosis [34]. However, the specific role of Met in OA chondrocytes ferroptosis and subchondral bone remains to be elucidated. The results of the present study demonstrated that Met may protect articular cartilage, as shown by inhibiting the degeneration of articular cartilage, and balancing the catabolism and anabolism of articular cartilage. Dixon et al. [10] regarded ferroptosis as a unique iron-dependent non-apoptotic form of cell death induced by Erastin. The results of the present study also demonstrated that Met may ameliorate Erastin-induced cartilage degeneration following the activation of chondrocyte ferroptosis by intra-articular injection of Erastin. Thus, we hypothesized that Met may delay cartilage degeneration in OA by targeting the occurrence of ferroptosis in chondrocytes. Subsequently, ferroptosis-related parameters were examined and the results of the present study demonstrated that Met may alleviate changes in the expression of ferroptosis-related proteins in mouse chondrocytes following Erastin treatment and also inhibit the occurrence of ferroptosis in mouse chondrocytes following the surgical induction of DMM. These findings also support our previous hypothesis that Met protects OA articular cartilage by inhibiting the occurrence of ferroptosis in OA chondrocytes.

Articular cartilage and subchondral bone act as a functional unit that cross talk each other to maintain the normal function of the knee joint. Drugs acting on the osteochondral functional unit are more effective than drugs acting solely on cartilage in treatment of OA [43]. The results of the present study demonstrated that Met not only acts on cartilage, but also exerts effects on the function of subchondral bone. Hu et al. [44] demonstrated that pathological manifestations of advanced OA occurred 8 weeks following surgery in a mouse OA model induced by DMM surgery. The results of the present study demonstrated that mice developed subchondral osteosclerosis 8 weeks after DMM surgery, and Met maintained the normal physiological structure of the subchondral bone by relieving the occurrence of advanced subchondral osteosclerosis. Moreover, the results of the present study demonstrated an increase in the expression of Runx2, which is closely associated with osteogenesis in the late stage of OA, while treatment with Met partially reduced the expression of Runx2 and inhibited the osteogenic enhancement of the subchondral bone in the late stage of OA. Ma et al. [24] demonstrated that OA progressed to advanced stages, not only with sclerosis of the subchondral bone, but also with an increase in atypical angiogenesis. These findings were verified in the present study, demonstrating that Met alleviated the sclerosis of subchondral bone in advanced stages of OA and inhibited the angiogenesis of abnormal subchondral bone. These findings further the current understanding of Met in the treatment of OA.

This study has some limitations. Notably, the present study was conducted in small mammals, and further investigations into the specific mechanisms by which Met targets ferroptosis to delay OA progression are required at the cellular level, as well as in large mammals, and in clinical trials using multicenter samples is required. Moreover, as gold standard for ferroptosis detection has not yet been established, only the induction of ferroptosis was determined in the chondrocytes of OA mice, preliminarily demonstrating that Met may inhibit ferroptosis and delay OA progression in vivo.

## Conclusion

The results of the present study demonstrated that Met attenuated the pathological manifestations of OA by inhibiting ferroptosis in OA chondrocytes, alleviating subchondral sclerosis in advanced OA, and reducing abnormal angiogenesis in subchondral bone in advanced OA. These findings further the current understanding of the therapeutic effects of Met in OA.

## Data Availability

The datasets used and/or analyzed during the current study are available from the corresponding author on reasonable request.

## Abbreviations

OA: Osteoarthritis
DMM: Destabilization of the medial meniscus
OARSI: Osteoarthritis Research Society International
HC: Hyaline cartilage
CC: Calcified cartilage
MMP: Metalloproteinase
Col II: Type II collagen
GPX4: Glutathione peroxidase 4
SLC7A11: Solute carrier family 7 member 11
ACSL4: Acyl-CoA synthase long-chain family member 4
BV/TV: Bone volume fraction
Tb. Sp: Trabecular separation
BMD: Bone mineral density
Runx2: Runt-associated transcription factor 2
CT: Computed tomography
